# Are Motor Imagery Ability scores related to cortical activity during gait imagery?

**DOI:** 10.21203/rs.3.rs-2777321/v1

**Published:** 2023-04-12

**Authors:** Martina Putzolu, Jessica Samogin, Gaia Bonassi, Carola Cosentino, Susanna Mezzarobba, Alessandro Botta, Laura Avanzino, Dante Mantini, Alessandro Vato, Elisa Pelosin

**Affiliations:** University of Genoa; KU Leuven; University of Genoa; University of Genoa; University of Genoa; IRCCS Ospedale Policlinico San Martino; University of Genoa; KU Leuven; Stratton VA Medical Center; University of Genoa

**Keywords:** Motor imagery, gait, motor imagery ability, event-related desynchronization (ERD), electroencephalography (EEG)

## Abstract

Motor imagery (MI) is the mental execution of actions without overt movements that depends on the ability to imagine. We explored whether this ability could be related to the cortical activity of the brain areas involved in the MI network. To this goal, brain activity was recorded using high-density electroencephalography (hdEEG) in nineteen healthy adults while visually imagining walking on a straight path. We extracted Event-Related Desynchronizations (ERDs) in the β band, and we measured MI ability via (i) the Kinesthetic and Visual Imagery Questionnaire (KVIQ), (ii) the Vividness of Movement Imagery Questionnaire-2 (VMIQ), and (iii) the Imagery Ability (IA) score. We then used Pearson’s and Spearman’s coefficients to correlate MI ability scores and average ERD power (*avgERD*). VMIQ was positively correlated with *avgERD* of frontal and cingulate areas, whereas IA SCORE was positively correlated with *avgERD* of left inferior frontal and superior temporal regions. Stronger activation of the MI network was related to better scores of MI ability evaluations, supporting the importance of testing MI ability during MI protocols. This result will help to understand MI mechanisms and develop personalized MI treatments for patients with neurological dysfunctions.

## Introduction

Motor imagery (MI) can be defined as a dynamic brain state during which representations of a given motor act are internally rehearsed in working memory without any overt motor output^1^. Over the past century, MI has been used to improve motor skills in athletes, musicians, and singers^2^ and promote motor learning processes and motor abilities in neurorehabilitation^3^. In addition, during the last two decades, with the increasing number of scientists involved in developing brain-computer interfaces (BCIs), motor imagery gained a spike of interest, being at the basis of many BCI-based prostheses or BCI systems designed to control external devices^4^.

So far, evidence supports the effectiveness of MI training in improving motor performances in healthy subjects, the elderly^5^, and individuals with neurological diseases^6^, even if to a lesser extent than motor practice^7^, in particular over time^8^. One reason that explains MI’s positive effect on motor skills learning is the significant overlap between the brain regions activated during motor imagery and motor execution (ME)^9–12^. Indeed, several neuroimaging and neurophysiological studies show that these two processes are part of the same motor representation system^11^. The lack of somatosensory input^7,8^ and movement inhibition in motor imagery^13^ can partially explain the more significant effect of ME compared to MI in motor skills learning.

Nevertheless, the neurophysiological mechanisms at the basis of MI effectiveness are still unclear, mainly due to the lack of accurate tools to measure the imagery ability of each individual^14^. Since imagination can differ in every person,^15,16^ researchers have developed methods for assessing the subject’s mental capacity to rehearse a specific movement or action. Most of these techniques are based on questionnaires where subjects are asked to score the imagery vividness from a first- or third-person perspective (visual MI) or the intensity of the sensation generated by the imagined action (kinesthetic MI). Mental chronometry^17^ is another technique that measures the difference between the duration of the imagined movement and its physical execution; a slight difference between the two durations reveals a well-trained motor imagery ability.

However, none of the tests and questionnaires provides knowledge about the relationship between the scores obtained from the subjects and the degree of cortical activation of the MI brain network during a motor imagery task. This lack of information leaves significant uncertainty in further considering the role of motor imagery practice in improving motor performance. Therefore, a more profound knowledge of the underlying mechanisms of MI is crucial for implementing its use in the clinical setting and developing personalized training protocols based on the subjects’ abilities.

This study aimed to investigate and quantify the relationship between MI ability and brain activity recorded during a MI task in healthy subjects. We assessed the correlation between the subjects’ scores obtained from MI ability tests and the electroencephalographic (EEG) brain activity recorded during a gait motor imagery task. As in our previous paper^18^, we focused our analysis of recorded high-density EEG signals (hdEEG) by measuring event-related desynchronization (ERD) in the beta frequency band (13 ÷ 30 Hz), applying a custom-developed pipeline for source localization. We hypothesized a correlation between the ability to imagine, measured using mental chronometry tests and questionnaires, and the amplitude of ERD in beta-frequency brain signals recorded from the cortical areas in the MI network.

## Methods

### Subjects

The data analyzed in this paper have been collected from 19 healthy adults, as described in a recent publication from our group^18^. We excluded from participation subjects (i) with previous experience with MI techniques or training, (ii) with a history of neurological diseases, or (iii) being treated with any medication that affected the central nervous system. We performed a sub-analysis after removing two subjects since the Imagery Ability Score was unavailable. The subjects provided informed written consent for participating in this study that was approved by the local ethical committee (Comitato Etico Regionale Liguria, Ref.1293 of September 12th, 2018) and conducted in conformity with the Declaration of Helsinki.

### Motor Imagery Ability assessment

To assess the subjects’ motor imagery ability, we collected each participant’s response to the Kinesthetic and Visual Imagery Questionnaire^19^ (KVIQ) and the Vividness of Movement Imagery Questionnaire-2 (VMIQ)^20^.

The KVIQ measures, on a five-point ordinal scale, the ability of the subject to mentally represent ten movements performed with all body segments. Subjects are required to imagine each of the ten movements and rate the clarity and intensity of the imagined movement from the first-person perspective.

The clarity of the image is evaluated using a visual sub-scale (KVIQ-v) from a score value equal to one, meaning “no image,” to five, meaning “image as clear as seeing.” The sensation intensity (KVIQ-k) is evaluated using a kinesthetic sub-scale from a score equal to one representing “no sensation” to five, meaning “as intense as executing the action The higher the score, the better the MI ability.

The VMIQ evaluates the imagery ability in three different conditions: internal (VMIQ-int) and external (VMIQ-ext) visual imagery, and kinesthetic imagery (VMIQ-k)^21,22^. We chose this questionnaire because each of the twelve actions to be imagined involves lower-limb movements or is related to gait tasks. The score ranges from 12 to 60; the lower the score, the better the personal estimation of the motor imagery ability. The VMIQ-int and the VMIQ-ext questionnaires consider a score equal to one point as “perfectly and clearly, as normal vision” and a score equal to five as “no image at all.” In the VMIQ-k questionnaire, a score of one means “perfectly and clearly, as the normal feel of movement” and a score of five points means “no feel at all”.

To evaluate MI ability, since we asked the participants to imagine themselves walking visually, we only used the scores from the VMIQ-int, VMIQ-ext, and the KVIQ-v.

To include an objective measure in our study, we calculated the Imagery Ability (IA) score defined by Beauchet and colleagues^23^. The IA score measures the temporal differences between performed and mentally simulated movements by combining two mental chronometry tests^24^: the Timed Up and Go^25^ (TUG) and the 10 Meters Walking Test^26^ (10MWT).

First, for each test a delta score (DS) was computed as follows:

$$DS=\frac{\left(T_{\text{real }}-T_{\text{imag}}\right)}{\left(T_{\text{real }}+T_{\text{imag}}\right)/2}\times 100$$

T_real_ is the time required to execute the task, and T_imag_ is the time needed for the mental simulation of the same task. Then, the IA score was calculated as the absolute value of the mean of the two delta scores^23^. The lower the IA score, the better the subject’s ability to imagine.

### Gait Motor Imagery Task

**Figure 1:**
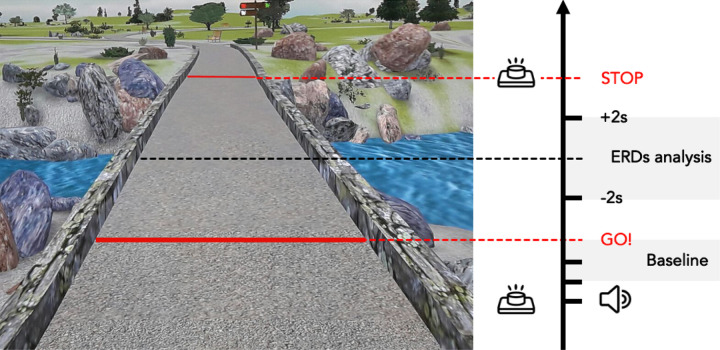
Motor imagery task. The picture shows a straight pathway; The black arrow indicates the task progression. The first red line (GO!) indicates the starting point of MI task; the second red line indicates the end of the MI task (STOP). The grey box “Baseline” indicates the time window selected as ERDs baseline analysis. The grey box “ERDs analysis” indicates the time window selected for ERDs analysis during MI task.

After the motor imagery ability assessment, the participants were seated in a quiet room in front of a screen displaying a picture representing a straight pathway with two red lines at the beginning and the end (Fig. 1). The task consists of visually imagining^24^ themselves walking on a straight path keeping their eyes open, starting from the first red line, and stopping when crossing the second one. To start the MI task, the subjects had to press a push-button and wait for a GO signal presented on the screen, preceded by a 4-second audio countdown (“3, 2, 1, GO”) before beginning to imagine. When they mentally crossed the second red line, they had to press the button again. The gait MI task consisted of three blocks of 10 trials each, in total 30 trials. During the inter-trial period, a fixation cross was presented on the screen for three seconds to prevent mental fatigue.

### EEG recording and processing

#### EEG data collection

We recorded the subjects’ brain activity using a high-density EEG (hdEEG) system equipped with 128 channels (Brain Products GmbH, Munich, Germany) with a sample rate of 1kHz. We positioned the active wet EEG electrodes according to the 5–10 system^27^, and used the FCz electrode as a physical reference to increase the signal-to-noise ratio as recommended by the manufacturer. We used a trigger box connected to the hdEEG system to record external events as the push-button presses and sync them with the brain signal. We recorded the vertical and horizontal electrooculographic signals (vEOG/hEOG) to identify and remove ocular-related artifacts from scalp registration. During each experimental session, we verified that the electrode impedance remained below 5kΩ throughout the session using the Brainvision Recorder software (Brain Products GmbH, Munich, Germany).

#### EEG data preprocessing

We first identified and corrected noisy channels by interpolating their time course from the adjacent channels. We fixed between 0 and 15 channels for each participant (median = 6; IQR = 6), mainly in the frontal area. Then we used the EEGlab software tool to band-pass filter the signals (1 ÷ 80 Hz) and reject ocular and muscular artifacts embedded in the data using Independent Component Analysis (ICA)^28^. Each independent component (IC) was classified according to three parameters: the correlation of the power of the IC with the power of vEOG and hEOG signals; the coefficient of determination obtained by fitting the IC power spectrum with a 1/f function; the kurtosis of the IC time-course^29,30^. We used the parameters’ thresholds as described in previous works^29,31^. The time courses of the ICs classified as bad were reconstructed at the channel level and subtracted from the hdEEG data. The number of artifactual ICs, identified and removed from the channel data, greatly varied among the datasets (median = 110; IQR = 65). Finally, we applied the average re-reference (AR) technique to re-reference the cleaned hdEEG recordings^32^.

#### EEG source localization

To localize the source of the EEG signals recorded from the scalp, we employed a robust and well-known automated pipeline^28,29,33,34^.

First, we built a head model based on a template magnetic resonance (MR) head image and template electrode positions^29,34,35^ to calculate the leadfield matrix. The MRI head image was segmented into 12 compartment^34^ and the template electrode positions were rigidly co-registered to the head contour (i.e., the outer layer of the skin compartment). Subsequently, by using Simbio (https://www.mrt.uni-jena.de/simbio), the numerical approximation of the whole-head volume conduction model was calculated as a finite element model^36^. At last, we created the leadfield matrix that expresses the scalp potentials corresponding to each source configuration. More details about head modeling have been previously reported^18^.

Finally, to estimate the brain activity of each voxel within the source space, the artifact-free re-referenced scalp hdEEG and the realistic head model were used as input to the exact low-resolution brain electromagnetic tomography algorithm (eLORETA)^37^.

### Event-Related Desynchronization analysis

For the following analysis, we selected 34 regions of interest (ROIs), included in the AAL brain atlas^38^, and previously associated with MI of walking^39–52^(Supplementary materials, Table S1). ROI coordinates were projected on the cortex of the template head. All voxels included in a spherical region with a 6mm radius and centered in the ROI coordinates were used to calculate the ROI activity and defined as ROI masks. We used the principal component of these voxels’ time courses to represent the ROI neural activity, and we analyzed these signals in the β frequency band (13 ÷ 30 Hz).

The event-related desynchronizations (ERDs) were assessed using source reconstructed data. We used the Short-Time Fourier Transform to perform a time-frequency decomposition on each voxel time course by applying a moving Hamming window of 2s, with 50% overlap between consecutive windows. Spectrograms were created in the frequency range 1 ÷ 80Hz, at steps of 1Hz, and epoched with a 4s time window. In our ERD analysis, we considered as *MI task* the 4s epoch (+ 2s; −2s) computed from the midpoint of each MI task and as *baseline* the 4s epoch centered 2s preceding the GO-signal in each trial.

The spectrogram epochs of MI tasks were averaged across trials, and the ERD intensity of each voxel was calculated as the percentage value of the relative difference between the epoch power at a given time point and the average baseline power for β band^28^. Then, we created the ERD spatial maps by averaging the time-frequency values corresponding to the relevant frequencies within the same range. As the final step, we converted the ERD maps reconstructed in individual spaces into MNI space^29,34,35^.

### Statistical analysis

For each participant and ROI mask, we selected the number of desynchronized (i.e., negative) grey matter voxels (i.e., dsv(i)) within the *i*^*th*^ ROI mask in the individual desynchronization map. These values were normalized on the total number of voxels within the corresponding mask with the formula:

$$ds\text{\%}(i)=100•\frac{dsv(i)}{\sum vox\left(mask_{i}\right)}$$

All the results were corrected for age. The individual mean amplitude of desynchronization (*avgERD*) within each ROI mask *(i)* was calculated as the average amplitude of the desynchronized voxels identified in the previous step.

We used Spearman’s rank correlation coefficient (ρ) to identify a possible relationship between the scores of visual imagery questionnaires (i.e., KVIQ-v, VMIQ-ext, and VMIQ-I) and the intensity of the desynchronization in each ROI of interest (i.e., *avgERD(i)*) and the Pearson’s correlation coefficient (R) for correlations between IA score and *avgERD(i)*. The significance of the tests was set at *p* < 0.05. All analyses were conducted with MATLAB ^®^ (R2018a, Math-Works, Natick, MA, USA).

## Results

Participants’ demographic and MI ability test scores are summarized in Table 1. Data from seventeen volunteers, ten females, mean age 35.88 ± 13.36 (SD) years (range 20–49), were entered in the statistical analysis. The mean (± SD) imagery ability scores of the KVIQ-v, VMIQ-ext and VMIQ-int were 43.41 ± 6.47, 23.82 ± 10.06, and 21.94 ± 9.27, respectively. The mean (± SD) delta score of TUG and 10MWT mental chronometry tests were of 0.17 ± 0.15 and 0.12 ± 0.08 seconds, respectively, determining a mean (± SD) IA score of 0.14 ± 0.08 seconds.

### Correlations

We found positive significant correlations between the power of desynchronizations in the β band (*avgERD(i)* data) and the MI ability scores (VMIQ-ext and IA score) in frontal areas, temporal areas, and the cingulate cortices. All significant correlations indicated that a better IA and VMIQ-ext score coincides to higher activation of areas involved in the MI network of gait.

**Figure 2:**
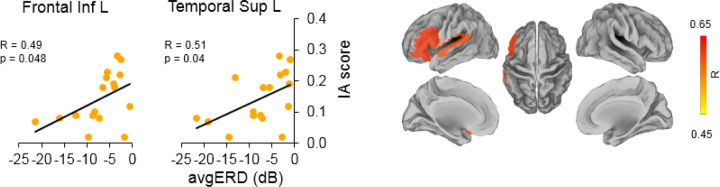
Correlations between IA score and *avgERD(i)* during MI of gait in β band. Significant correlations graphs between activations of (ii) the left inferior frontal and (ii) the left superior temporal areas with IA SCORE are shown on the left side of the picture. On the right side of the figure, magnitude of Pearson’s correlation coefficient (R) is visible in lateral, medial and dorsal views of brain maps. L = Left, Inf = Inferior, Sup = Superior, IA = Imagery Ability

**Figure 3:**
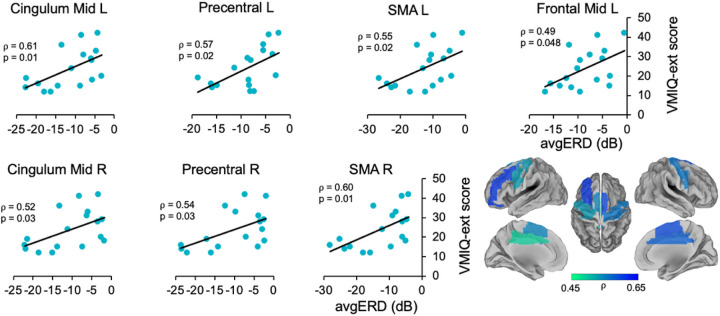
Correlations between VMIQ-ext score and *avgERD(i)* during MI of gait in β band. The left side of the image displays significant correlations between the activations of the left middle frontal area, middle cingulum, precentral regions, and supplementary motor areas with VMIQ-ext. On the right side of the figure, magnitude of Spearman’s rank correlation coefficient (ρ) is shown in lateral, medial and dorsal views of brain maps. Mid = Middle, L = Left, R = Right, SMA = Supplementary Motor Area, VMIQ-ext = Vividness of Movement Imagery Questionnaire - External

Precisely, a significant relationship was found with VMIQ-ext scores in the bilateral middle cingulum (left: =0.61, *p* = 0.01; right: =0.52, *p* = 0.03). In frontal regions, we found significant correlations in the left inferior frontal region with the IA score (R = 0.49, *p* = 0.048) and in the left middle frontal area with VMIQ-ext score ( =0.49, *p* = 0.048). Moreover, in the bilateral precentral area (left: =0.57, *p* = 0.02; right: =0.54, *p* = 0.03), and in the Supplementary Motor Area (SMA), bilaterally (left: =0.55, *p* = 0.02; right: =0.60, *p* = 0.01), we found a significant correlation between *avgERD(i)* and VMIQ-ext. Lastly, a significant correlation with IA score was found in the left superior temporal area (R = 0.51, *p* = 0.04). No significant relationships were found when correlating *avgERD(i)* with KVIQ-v and VMIQ-int scores. Correlations between the power of desynchronization and MI ability scores are shown in Fig. 2 (*avgERD*-IA SCORE) and Fig. 3 (*avgERD*-VMIQ-ext). The left side of the figures displays scatter plots, whereas the right side shows the magnitude of Pearson’s and Spearman’s rank correlation coefficients in the lateral, medial and dorsal views of brain maps.

## Discussion

In this study, we aimed to explore whether MI ability was associated with the cortical activity of brain regions of the MI network in a group of healthy subjects during a gait imagery task. Precisely, results for the vividness of imageries tested with KVIQ and VMIQ questionnaires, and the ability of imagery, measured using mental chronometry assessments were correlated with power changes in β band, recorded with hdEEG.

The main result of this study was that we found a significant relationship between the power of the activity in the areas already shown^37–50^ to be involved in MI of gait and the MI ability scores.

Precisely, a positive correlation was detected between VMIQ-ext scores and ERDs β band power of frontal and cingulate areas, and between IA scores and the power of activity of the left inferior frontal and superior temporal regions.

To date, only few studies tried to investigate whether the individual ability to imagine vividly was associated with distinctive brain activity patterns^51,53,54^. The first paper, published in 1992 by Charlot and co-workers^53^, measured brain activity in healthy undergraduate volunteers, classified, as “high” and “low” imagers, based on the score of two clinical tests (i.e., the Minnesota Paper Form Board and the Mental Rotations Test), during a visual imagery task consisting in a mental representation and exploration of an imaginary island. Using regional cerebral blood flow (CBF) imaging, they found that low imagers had a widespread CBF increase, whereas high imagers showed a more focal activation^53^. This finding was explained by authors hypothesizing a low cognitive functions differentiation in bad imagers and, conversely, a more differentiated cognitive architecture in skilled imagers. Later, differences in brain activity, using functional magnetic resonance imaging (fMRI), were investigated in participants showing high or poor MI ability, during both physical execution and mental imagery of finger movements^54^. Results revealed that good imagers had a higher bilateral activation in the premotor, parietal regions, known to have crucial role in the MI network, with respect to bad imagers. By contrast, participants with poor MI ability manifested greater posterior cingulate, orbitofrontal areas, and cerebellum activations, possibly reflecting a compensatory mechanism to counteract difficulties in creating a vivid representation of sequential movements.

Concerning evidence investigating differences among subjects with good and poor MI ability during MI of gait, it has been reported that imagery capacities do influence functional brain activity even during the imagination of a simple and well automatized motor task. Meulen and collaborators^51^, in fact, found that participants with good MI ability had a higher cortical activation in the primary motor cortex, the prefrontal cortex, thalamus, and cerebellum with respect to those with lower imagery performance.

In line with these results, also here we found that MI ability level influenced cortical recruitment specifically in those areas which are particularly involved in the MI neural network. Precisely, a positive correlation was found between the MI ability test scores and the left inferior and middle frontal areas, the precentral regions, and the SMAs, suggesting that the better the IA, the more the involvement of these areas. Frontal activity is known to be crucial for MI and especially for MI of gait, supporting the fact that gait is no longer considered a simple and automatic motor action. Indeed, various cognitive functions (such as attention and visuo-spatial abilities) are involved during walking, especially during complex tasks, thus justifying the intervention of frontal regions for being in charge of the higher-order cognitive control of gait^55^.

Temporal areas were involved through the left superior temporal region activation, showing a positive correlation with IA SCORE. Temporal regions are usually recognized to participate in allocentric processing, fundamental for activities involving spatial memory and navigation. Even if also egocentric processing is implicated in navigation, allocentric processing might help in maintaining a cognitive representation of the environment by updating our own location within it and in avoiding cumulative errors associated with egocentric representation^56^. This assumption might explain the higher activation of temporal regions in participants who had a better objective ability in performing MI, measured via mental chronometry.

Finally, scores obtained by volunteers during the third person VMIQ test, were significantly positively correlated with *avgERD(i)* of middle cingulum. This brain region is recognized to be part of the MI network, and its activity results to be crucial for performing MI, specifically when considering MI of usual gait^52^.

It is worthy to note that no significant correlations were found between brain activity and scores of the first-person visual perspective of KVIQ (i.e., KVIQ-v) and of the of VMIQ (i.e., VMIQ-int). This could be related to the nature of our gait imagery task, where the external strategy might fit better when observing a path and imagining of moving forward.

A possible explanation might be represented by the different brain processes that took place when subjects have to execute visual MI in first person respect to a third-person perspective. Indeed, it was recently speculated that first-person imagery uses a bottom-up strategy, thus taking into account actions and reactions to concrete aspects of the imagined environment, whereas third-person imagery uses a top-down strategy due to the integration of the MI event with its wider context, including experience of other events beyond the main one^57^.

Finally, no results revealed a negative correlation between MI ability tests and MI network activity, supporting the hypothesis that a finest MI ability is associated with a higher recruitment of regions involved in MI network.

Several limitations of the study deserve attention. First, the small sample size lessened the strength of our results. Second, leg muscle activity was not recorded during the hdEEG registration. Nonetheless, previous studies showed that EMG activity of distal leg muscles recorded during seated position decreased, while during standing gait MI tasks led to a facilitatory effect on proximal lower limb muscle activity^58,59^. According to these findings we might suppose an irrelevant effect of leg muscle activity on EEG data acquisition. Third, even though cerebellar activity has been linked to MI cortical network, data consistency of hdEEG in detecting signals from the cerebellum is still up for debate^18^.

## Conclusions

In this study, we investigated whether MI ability could be associated to neural activity during a gait imagery task performed by healthy subjects during hdEEG registration. Our findings confirmed that scores obtained in the MI ability tests were related to the activations in the MI network, supporting the importance of testing MI ability with subjective questionnaires and mental chronometry in subjects involved in research and clinical protocols. This will help, first, in deeply understanding the neural mechanisms underpinning MI and, second, in developing tailored physiotherapy protocols based on the IA of patients. Nevertheless, we know that MI is a complex task, and several other aspects should be considered besides subjective questionnaires and chronometry performance to measure MI ability in healthy subjects better. Hence, future studies are needed to confirm our findings and to elucidate whether the relationships between MI ability and cortical activations could be influenced both by participants’ previous experience and the type of motor task (e.g., tasks based on subjects’ motor repertoire and more complex tasks, such as dual-task gait).

## Figures and Tables

**Table 1:** Demographic characteristics and behavioral data

Gender (n. female, %)	10 (58.82%)
Age (years)	35.88 ±13.36
Education (years)	19 ±2.21
IA (score)	0.14 ± 0.08
TUG (delta)	0.17 ± 0.15
10MWT (delta)	0.12 ± 0.08
KVIQ-v (score)	43.41 ± 6.47
VMIQ-ext (score)	23.82 ±10.06
VMIQ-int (score)	21.94 ± 9.27

Values are presented as mean ± standard deviation. IA, Imagery Ability; TUG, Timed Up and Go test; 10MWT, 10 Meters Walking Test; KVIQ, Kinesthetic and Visual Imagery Questionnaire; v, Visual; VMIQ, Vividness of Movement Imagery Questionnaire-2; ext, External; int, Internal
